# Geometry-Driven Phase Error Estimation for Azimuth Multi-Channel SAR via Global Radar Landmark Control Point Library

**DOI:** 10.3390/s26051622

**Published:** 2026-03-05

**Authors:** Tingting Jin, Zheng Li, Feng Wang, Hui Long

**Affiliations:** 1 Key Laboratory of Technology in Geo-Spatial Information Processing and Application System, Chinese Academy of Sciences, Beijing 100190, China; lizheng@aircas.ac.cn (Z.L.); wangfeng@aircas.ac.cn (F.W.); longhui@aircas.ac.cn (H.L.); 2 Aerospace Information Research Institute, Chinese Academy of Sciences, Beijing 100094, China; 3 Key Laboratory of Target Cognition and Application Technology (TCAT), Beijing 100190, China

**Keywords:** synthetic aperture radar (SAR), high-resolution wide-swath (HRWS), azimuth multichannel SAR, inter-channel phase inconsistency, frequency-domain correlation, global radar landmark control point (GRL-CP) library

## Abstract

**Highlights:**

**What are the main findings?**

- A geometry-driven inter-channel phase error estimation framework is developed by introducing a Global Radar Landmark Control Point Library (GRL-CP) and automatic range–Doppler-based control point activation, eliminating scene-dependent and manual target selection.
- A frequency-domain correlation strategy based on activated radar landmarks enables robust and accurate phase error estimation, outperforming conventional correlation and subspace-based methods in complex multi-scene environments.

**What is the main implication of the main findings?**

- The proposed framework enables fully automated, reliable, and scalable phase calibration for high-resolution wide-swath SAR systems, supporting operational and large-scale multi-channel SAR data processing.

**Abstract:**

Azimuth multi-channel synthetic aperture radar (SAR) is a core technology for achieving high-resolution wide-swath (HRWS) imaging. However, inter-channel phase inconsistency causes image amplitude distortion and phase accuracy degradation, which severely affects subsequent applications. Existing phase error estimation methods face specific limitations: the performance of subspace-based approaches degrades in complex scenes due to unreliable covariance matrix estimation, while conventional frequency-domain correlation methods rely on manual selection of strong scatterers, introducing inefficiency and subjectivity that precludes autonomous deployment. To address these issues, this paper proposes a geometry-driven inter-channel phase error estimation framework based on Global Radar Landmark Control Point Library (GRL-CP). The proposed framework replaces scene-dependent target selection with geometric-prior-driven control point activation. The GRL-CP library stores only the geodetic coordinates and scattering stability attributes of globally persistent radar landmarks, rather than image patches. For a new SAR acquisition, the echo position of these landmarks are predicted using a range–Doppler geometric model, enabling fully automatic and reliable control point activation. Based on the activated radar landmarks, inter-channel phase error is estimated using a frequency-domain correlation scheme. Experimental results on multi-channel spaceborne SAR datasets demonstrate that the proposed method achieves improved stability and accuracy under complex terrain scenarios.

## 1. Introduction

Spaceborne synthetic aperture radar (SAR) has become an indispensable Earth observation technology due to its all-weather, all-day imaging capability and strong penetration performance. It plays a vital role in a wide range of applications, including environmental monitoring, disaster assessment, urban mapping, and climate change analysis [1,2,3,4,5]. As the demand for timely and large-scale observation continues to grow, achieving high-resolution wide-swath (HRWS) imaging has become a fundamental objective of modern spaceborne SAR sensor.

In conventional single-channel SAR systems, azimuth resolution and swath width are inherently constrained by the pulse repetition frequency (PRF). A high PRF enables high resolution but limits swath coverage, while a low PRF supports wide-swath imaging at the cost of azimuth resolution. This fundamental constraint hinders single-channel SAR from achieving HRWS imaging.

Azimuth multichannel SAR technology effectively alleviates this contradiction by employing multiple azimuth-displaced receive channels to increase the effective azimuth sampling rate without raising the PRF, thereby enabling HRWS imaging [6,7,8]. However, the multichannel imaging process is highly sensitive to inter-channel inconsistencies caused by electronic hardware variations, antenna array imperfections, and satellite platform attitude fluctuations. These inconsistencies introduce various error sources, including amplitude errors, phase errors, sampling delay errors, and antenna phase center position errors, which degrade imaging quality by generating false targets, reducing coherence, and impairing quantitative SAR applications [9,10,11,12,13]. Among these error sources, inter-channel phase errors are particularly critical: amplitude mismatches can be largely compensated through pre-channel equalization, whereas phase errors directly affect azimuth signal reconstruction and image fidelity.

Extensive studies have investigated the impact of inter-channel errors and corresponding mitigation strategies in azimuth multichannel SAR systems [6,7,8,9,10,11,12,13,14,15,16,17]. Existing inter-channel phase error estimation methods can generally be classified into three categories: correlation-based methods in the time or frequency domain, subspace-based methods relying on signal or noise subspace analysis [3,18], and iterative optimization-based methods such as adaptive least squares and minimum entropy approaches [15,19]. In [14], a time-domain correlation approach was introduced, which estimates the channel imbalance by calculating the cross-correlation of the raw echo data between different channels. While this approach is favored for its computational simplicity and ease of hardware implementation, it typically requires the presence of manually selected stable and isolated strong scatterers to ensure estimation accuracy. This reliance on specific target features significantly limits its automation and makes it highly scene-dependent. In [18], the authors proposed an orthogonal subspace-based method that estimates phase errors by performing eigenvalue decomposition on the signal covariance matrix to separate the signal and noise subspaces. The method’s core advantage lies in its high estimation precision, as it avoids the need for manual scatterer selection by exploiting the orthogonality between the steering vector and the noise subspace. However, its performance is highly sensitive to the accuracy of the covariance matrix, which often requires a large number of independent and identically distributed (i.e., homogeneous) samples, making it less robust in complex or highly heterogeneous urban scenes. In [19], an iterative optimization method based on the minimum entropy criterion was developed. This approach treats the phase error as an optimization variable and iteratively searches for the phase values that minimize the image entropy, thereby achieving auto-focusing and channel calibration simultaneously. Although this method can achieve superior image quality in complex environments without requiring prior scene information, it suffers from high computational complexity and potential convergence issues, which may hinder its application in large-scale data processing. Correlation approaches are computationally efficient and easy to implement, which makes them attractive for engineering applications; however, they typically rely on manually selected strong or isolated scatterers, resulting in limited automation and strong scene dependence. Subspace-based methods are sensitive to noise and modeling inaccuracies, particularly in complex or heterogeneous scenes, while iterative optimization-based methods often suffer from high computational complexity and convergence issues. Consequently, achieving automated, accurate, and scene-independent inter-channel phase error estimation remains a key challenge for spaceborne multichannel HRWS SAR systems. Related studies on channel imbalance, phase noise compensation, quantization effects, and image-domain calibration further highlight the complexity of this problem and the limitations of existing solutions [20,21,22,23,24,25,26,27,28,29,30,31,32,33]. Recent researches have further advanced these efforts, including range-dependent channel calibration for HRWS SAR imagery [34], azimuth-variant attitude error correction [35], and real-time channel error calibration algorithms [36].

In addition, several phase correction and autofocusing techniques have been developed for ISAR and non-cooperative moving targets, including pulse-to-pulse phase correction and contrast-maximization-based approaches [37,38,39]. These methods primarily address motion-induced pulse-to-pulse phase errors caused by target dynamics. In contrast, the present work focuses on systematic inter-channel phase errors in azimuth multichannel SAR systems under stationary-scene assumptions. The underlying problem formulation and estimation framework are therefore different.

A common characteristic of existing phase error estimation methods is their reliance on scene-dependent image-domain features, such as strong or isolated scatterers. The availability and reliability of such features vary significantly across different imaging scenes, acquisition geometries, and sensor configurations, which restricts the robustness and generalization capability of these methods. Since inter-channel phase errors stem from geometric and hardware factors rather than image content, geometry-based prior information can offer a more stable reference for phase error estimation.

Motivated by this observation, this work reinterprets long-term stable strong scatterers as geometric radar landmarks and exploits their geodetic coordinates as prior information. By constructing a global library of radar landmark control points, reliable phase estimation references can be automatically activated in new SAR acquisitions through geometric prediction, without relying on scene-dependent target selection. This geometry-driven strategy replaces heuristic image-domain selection with a geometric-prior-based control point activation mechanism, improving automation and cross-scene consistency.

The main contributions of this work are summarized as follows:

(1) A geometry-driven inter-channel phase error estimation framework is proposed to replace scene-dependent strong scatterer selection with a geometric-prior-based radar landmark framework.

(2) A global radar landmark control point library (GRL-CP library) is constructed, which stores only georeferenced, long-term stable radar landmarks and supports cross-scene and cross-sensor phase error estimation.

(3) A geometry-driven control point activation mechanism is developed to automatically associate radar landmarks with SAR echoes via a range–Doppler geometric model.

(4) Experimental results on spaceborne SAR sensor datasets verify the accuracy and stability of the proposed method.

The remainder of this paper is organized as follows. Section 2 presents the signal model of spaceborne azimuth multichannel SAR. Section 3 introduces the proposed geometry-driven inter-channel phase error estimation method. Section 4 describes the experimental datasets and presents the corresponding results. Section 5 discusses the performance of the proposed approach. Section 6 concludes the paper.

## 2. Signal Model

### 2.1. Multi-Channel SAR Signal Model

**Figure 1:**
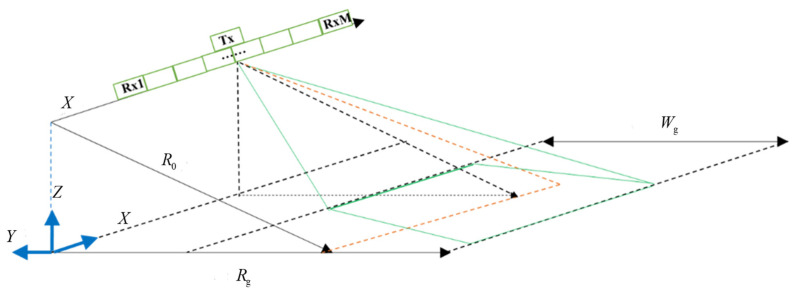
Schematic of azimuth multi-channel SAR imaging geometric model.

The multi-channel SAR signal model adopted here follows the classical formulation widely used in SAR literatures [40], and is briefly summarized for completeness. Figure 1 illustrates a geometric model of azimuth multi-channel SAR imaging, where the satellite platform is moves along the *x*-axis, the *z*-axis points away from the center of the earth, and the three coordinate axes form a three-dimensional Cartesian coordinate system. The satellite platform speed is $v_{s}$, the closest slant distance from the satellite platform to the center of the observation scene is $R_{0}$, the transmit pulse is emitted from the phase center of the full aperture, and each sub-antenna receives the echo signal with a slightly different time delay due to its spatial separation and slant range difference. The transmitting antenna is assumed to be co-located with the reference phase center of the receiving array, ensuring consistent signal geometry for phase modeling. Assuming that the length of the entire antenna is $L_{a}$, let M denote the total number of receiving channels, and m=1,2,…,M denote the channel index. Due to the uniform distribution of sub-apertures, the phase center interval between adjacent beams is $d=L_{a}/M$. Assuming that when the azimuth time is 0, the coordinates of the phase center of the entire antenna in the azimuth-slant range plane are $\left(x_{0},0\right)$, and the actual phase center coordinates of each sub-beam are $\left(x_{0}+∆x_{m},0\right)$, then

(1) $$∆x_{m}=\left(m-\left(M+1\right)/2\right)d,\;\;m=1,2,3,\cdots M$$

In the single-transmit single-receive mode, the static scene echo signal received by the reference channel $s_{0}\left(\tau ,\eta \right)$ and the signal received by the m-th channel $s_{m}\left(\tau ,\eta \right)$ in the one-transmit multiple-receive mode can be expressed as:

(2) $$s_{0}\left(\tau ,\eta \right)=\iint \sigma \left(x,r\right)g\left(\eta \right)h\left(\tau -\frac{2R_{T}\left(x,r,\eta \right)}{c}\right)\exp\left(-j\frac{2\pi R_{T}\left(x,r,\eta \right)}{\lambda }\right)dxdr$$

(3) $$\begin{array}{c} s_{m}\left(\tau ,\eta \right)=\iint \sigma \left(x,r\right)g\left(\eta \right)h\left(\tau -\frac{R_{T}\left(x,r,\eta \right)+R_{m}\left(x,r,\eta \right)}{c}\right)\\ \cdot \exp\left(-j\pi \frac{R_{T}\left(x,r,\eta \right)+R_{m}\left(x,r,\eta \right)}{\lambda }\right)dxdr \end{array}$$

where τ,η denotes range time and azimuth time respectively, c is the speed of light, λ is the radar wavelength, $\sigma \left(x,r\right)$ is the complex scattering coefficient at coordinate $\left(x,r\right)$ in the azimuth-slant range plane, $g\left(\eta \right)$ is the antenna pattern in azimuth direction, and $h\left(\tau \right)$ is transmit pulse. $R_{T}\left(x,r,\eta \right)$ and $R_{R}\left(x,r,\eta \right)$ are the slant distances between the reference channel, the m-th channel and the scene slant distance plane coordinates:

(4) $$R_{T}\left(x,r,\eta \right)=\sqrt{{\left(v_{s}\eta +x_{0}-x\right)}^{2}+r^{2}}$$

(5) $$R_{R}\left(x,r,\eta \right)=\sqrt{{\left(v_{s}\eta +x_{0}+∆x_{m}-x\right)}^{2}+r^{2}}$$

Then the echo history of each sub-beam $R_{m}\left(x,r,\eta \right)$ is the sum of the transmitting history and the receiving history:

(6) $$\begin{array}{cc} R_{m}\left(x,r,\eta \right) & =\sqrt{{\left(v_{s}\eta +x_{0}-x\right)}^{2}+r^{2}}+\sqrt{{\left(v_{s}\eta +x_{0}+∆x_{m}-x\right)}^{2}+r^{2}}\\ & =\sqrt{r^{2}+{\left(v_{s}\eta +x_{0}-x-\frac{∆x_{m}}{2}+\frac{∆x_{m}}{2}\right)}^{2}}+\sqrt{r^{2}+{\left(v_{s}\eta +x_{0}-x-\frac{∆x_{m}}{2}-\frac{∆x_{m}}{2}\right)}^{2}} \end{array}$$

Let $f\left(\eta \right)=v_{s}\eta +x_{0}-x-\frac{∆x_{m}}{2}$, substitute into the above formula

(7) $$R_{m}\left(x,r,\eta \right)=\sqrt{r^{2}+{\left(f\left(\eta \right)+\frac{∆x_{m}}{2}\right)}^{2}}+\sqrt{r^{2}+{\left(f\left(\eta \right)-\frac{∆x_{m}}{2}\right)}^{2}}$$

Since $∆x_{m}$ is much smaller than the slant distance r and $f\left(\eta \right)$, the second-order Taylor series expansion is performed at $∆x_{m}=0$:

(8) $$\begin{array}{cc} R_{m}\left(x,r,\eta \right) & \approx 2\sqrt{r^{2}+f{\left(\eta \right)}^{2}}+\frac{r^{2}}{{\left(r^{2}+f{\left(\eta \right)}^{2}\right)}^{3/2}}∆x_{m}^{2}\\ & =2R_{T}\left(x,r,\eta -\frac{∆x_{m}}{2v_{s}}\right)+\frac{r^{2}}{{\left(r^{2}+f{\left(\eta \right)}^{2}\right)}^{3/2}}∆x_{m}^{2} \end{array}$$

It can be concluded that $s_{m}\left(\tau ,\eta \right)$ from $s_{0}\left(\tau ,\eta \right)$ by applying a certain time delay and phase shift. The time delay is $∆\eta_{m}={∆x_{m}/2v}_{s}$, the phase shift is

(9) $$\varphi_{m}\left(\eta \right)=-\frac{2\pi }{\lambda }\frac{r^{2}}{{\left(r^{2}+{\left(v_{s}\eta +x_{0}-\frac{∆x_{m}}{2}\right)}^{2}\right)}^{3/2}}∆x_{m}^{2}$$

After compensating the constant phase, the relationship between the m-th channel echo and the reference channel echo is:

(10) $$s_{m}\left(\tau ,\eta \right)\approx s_{0}\left(\tau ,\eta +∆\eta_{m}\right)$$

Therefore, the echoes from different channels have a fixed time delay in azimuth time, and only differ in linear phase in the Doppler domain, which can be expressed as

(11) $$s_{m}\left(\tau ,f_{a}\right)\approx s_{0}\left(\tau ,f_{a}\right)e^{j2\pi ∆\eta_{m}f_{a}}$$

where $f_{a}$ is the azimuth frequency, each Doppler unit contains countless scene echoes with the same cone angle, and the relationship between the cone angle and the azimuth frequency is:

(12) $$f_{a}=\frac{2v_{s}}{\lambda }\sin\phi_{c}\left(f_{a}\right)$$

where $\phi_{c}$ represents the beam cone angle corresponding to the stationary target. In other words, the space-time spectrum of the Doppler frequency and the sine value of the cone angle is a straight line with a constant slope. However, due to azimuth undersampling in each channel, the Doppler domain signal becomes ambiguous, meaning that a single cone angle may correspond to multiple Doppler frequencies. Let the Doppler ambiguity number be 2N + 1 (N is a positive integer representing the Doppler ambiguity order; for convenience, it is set to an odd number), which is the ratio of the Doppler bandwidth to the pulse repetition frequency of the echo signal of each channel. Then the actual range time domain-azimuth frequency domain echo of the m-th channel is expressed as

(13) $$S_{m}\left(\tau ,f_{a}\right)\approx \sum_{n=-N}^{N}S_{0}\left(\tau ,f_{a}+n\cdot f_{p}\right)e^{j2\pi ∆\eta_{m}\left(f_{a}+n\cdot f_{p}\right)}$$

where $f_{p}$ denotes the pulse repetition frequency.

### 2.2. Fundamentals of Orthogonal Subspace-Base Calibration

To provide a theoretical basis for comparison, the fundamentals of the orthogonal subspace method are briefly introduced. This method assumes that the inter-channel amplitude and phase inconsistencies are constant for a given data acquisition. In the presence of channel errors, the covariance matrix of the received multi-channel signal $\hat{R}$ can be expressed as:

(14) $$\hat{R}=E\left\{S\left(\tau ,f_{\eta }\right)S^{H}\left(\tau ,f_{\eta }\right)\right\}$$

where $S\left(\tau ,f_{\eta }\right)$ represents the echo signal vector in the range–Doppler domain. By performing eigenvalue decomposition on the estimated covariance matrix $\hat{R}$, it can be decomposed into signal and noise subspace:

(15) $$\hat{R}=U_{S}\Sigma_{S}U_{S}^{H}+U_{N}\Sigma_{N}U_{N}^{H}$$

where $U_{S}$ is the signal subspace spanned by the eigenvectors corresponding to the 2I+1 largest eigenvalues (where I denotes the Doppler ambiguity order), $\Sigma_{S}$ is the diagonal matrix containing the 2I+1 largest eigenvalues, $U_{N}$ is the noise subspace spanned by the remaining eigenvectors, and $\Sigma_{N}$ contains the remaining noise eigenvalues.

According to the subspace theory, the steering vector of the signal is orthogonal to the noise subspace. Therefore, the inter-channel phase error vector γ can be estimated by solving the following optimization problem:

(16) $$\underset{\gamma }{min}\gamma^{H}\left[\sum_{i=-I}^{I}Q_{i}^{H}U_{N}U_{N}^{H}Q_{i}\right]\gamma s.t.\gamma^{H}w=1$$

where $Q_{i}=diag\left\{a_{i}\right\}$ and w is a reference channel weight vector, $a_{i}$ denotes the steering vector corresponding to the i-th Doppler ambiguity. The optimal solution for the phase error is obtained by finding the eigenvector corresponding to the minimum eigenvalue of the noise subspace projection matrix.

## 3. Proposed Method

**Figure 2:**
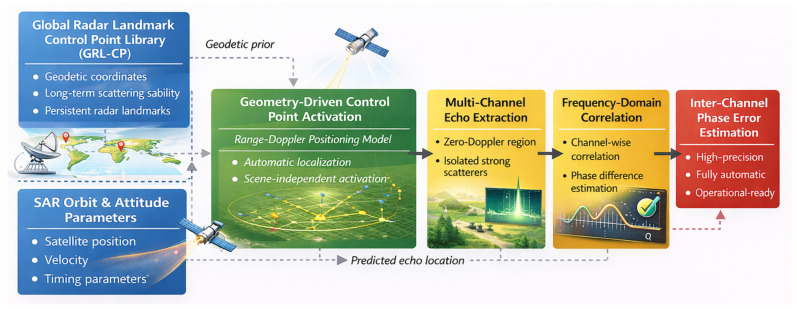
Overall framework of the proposed geometry-driven inter-channel phase error estimation method. A global radar landmark control point library provides geodetic and scattering stability priors. Using SAR orbit and attitude parameters, reliable control points are automatically activated via a range–Doppler geometric model. Multi-channel echoes are then extracted around zero Doppler, and inter-channel phase errors are estimated using frequency-domain correlation.

The proposed geometry-driven framework utilizes inter-channel phase error estimation based on a geometric-prior-driven approach. As illustrated in Figure 2, the methodology includes three main steps:

Construction of GRL-CP Library: A global radar landmark control point library is built to store the geodetic coordinates and scattering stability attributes of isolated strong scatterers.

Control Point Activation: For any given SAR acquisition, landmarks are automatically activated and their echo positions are localized using SAR positioning geometry and a range–Doppler model.

Phase Error Estimation: Inter-channel phase errors are robustly estimated through frequency-domain correlation using the activated scatterers near the zero-Doppler region.

By integrating geometric priors, SAR positioning models, and signal-domain correlation, this framework establishes a fully automatic and operationally robust phase error estimation method for azimuth multi-channel SAR systems.

### 3.1. Construction of Global Radar Landmark Control Point Library

The proposed GRL-CP is a georeferenced control point database. Each entry stores the geodetic coordinates and scattering stability attributes of globally persistent strong scatterers, rather than image patches. The underlying assumption is that certain man-made structures (e.g., corner reflectors, transmission towers, bridges, and metallic buildings) exhibit long-term, stable high radar cross sections, and thus remain strong scatterers across different SAR acquisitions and viewing geometries.

The construction procedure is summarized as follows.

(1) Candidate control point mining from historical SAR images

Let $I_{k}\left(x,y\right)$ denote radiometrically calibrated SAR intensity image acquired at time k. Strong scatterer candidates are detected by a local contrast test:

(17) $$\left(x_{i},y_{i}\right)\in C_{k}\;\;\;\;if\;\;\;\frac{I_{k}\left(x_{i},y_{i}\right)}{\mu_{bg}\left(x_{i},y_{i}\right)}\geq T$$

where $\mu_{bg}\left(x_{i},y_{i}\right)$ represents the local background power estimated within a surrounding window and T is the detection threshold, typically set to 10–15 dB.

Each candidate is subsequently geocoded using the corresponding imaging geometry to obtain its geodetic coordinates:

(18) $$P_{i}=\left(lat_{i},lon_{i},h_{i}\right)$$

where $P_{i}$ denotes the geodetic position of the i-th activated radar landmark, expressed in geographic coordinates. Specifically, $lat_{i}$ and $lon_{i}$ represents the latitude and longitude in the WGS-84 coordinate system, respectively, and $h_{i}$ denotes the ellipsoidal height above the reference ellipsoid.

(2) Temporal stability screening

To ensure long-term persistence, a valid control point must be observed in multiple independent acquisitions. Specifically, a candidate point must appear in at least M acquisitions:

(19) $$count\left(P_{i}\right)\geq M,M=3∼5$$

Furthermore, its backscattering response must exhibit low temporal fluctuation. This requirement is enforced by constraining the coefficient of variation of the backscattering coefficient $\sigma^{0}$:

(20) $$\frac{\sigma_{t}\left(\sigma_{i}^{0}\right)}{\mu_{t}\left(\sigma_{i}^{0}\right)}<\eta ,\eta \approx 0.3$$

where $\mu_{t}\left(\sigma_{i}^{0}\right)$ and $\sigma_{t}\left(\sigma_{i}^{0}\right)$ denote the temporal mean and standard deviation of $\sigma^{0}$ over all available acquisitions. This criterion effectively filters out temporally unstable or decorrelated scatterers.

(3) Spatial isolation verification

To avoid distributed or clustered scatterers that may suffer from mutual interference under different resolutions and viewing geometries, a spatial isolation constraint is imposed. For each candidate control point, the minimum separation from neighboring points must satisfy:

(21) $$\underset{j\neq i}{\min}\left\|r_{i}-r_{j}\right\|>d_{iso},\;\;\;\;d_{iso}=30∼50\;m$$

where $r_{i}$ denotes the Cartesian position of $P_{i}$ in an Earth-centered coordinate system. This constraint ensures that the selected control point remains isolated across different SAR systems and imaging configurations.

(4) GRL-CP definition and structure

After the above screening steps, the GRL-CP library is defined as:

(22) $$L={\left\{p_{i},M_{i}\right\}}_{i=1}^{N}$$

where N denotes the number of globally distributed, stable radar landmarks. Each library entry contains only geometric and statistical attributes, without storing image patches:

(23) $$M_{i}=\left\{lat_{i},lon_{i},h_{i},\mu_{t}\left(\sigma^{0}\right),\sigma_{t}\left(\sigma^{0}\right),\lambda_{i},\phi_{i},\theta_{d,i}\right\}$$

where $\lambda_{i}$, $\phi_{i}$ and $\theta_{i}$ (wavelength, polarization and incidence angle) denotes the characteristic imaging or scattering parameters associated with the control point relating to the scattering mechanism.

(5) Usage logic in new scenes

Given a new SAR acquisition, each control point $p_{i}\in L$ is automatically activated by predicting its expected echo position using the range–Doppler geometric model:

(24) $$\left(r_{i},a_{i}\right)=f_{RD}\left(p_{i}\right)$$

where $f_{RD}\left(\cdot \right)$ maps geodetic coordinates to range and azimuth sample indices based on the sensor orbit and imaging parameters. The local echo segment centered at $\left(r_{i},a_{i}\right)$ is then extracted and used for subsequent inter-channel phase error estimation.

### 3.2. Geometry-Driven Control Point Activation and Echo Localization

Given a new SAR acquisition, control points in the GRL-CP library are automatically activated using geometric prediction without any scene-dependent target detection.

**Figure 3:**
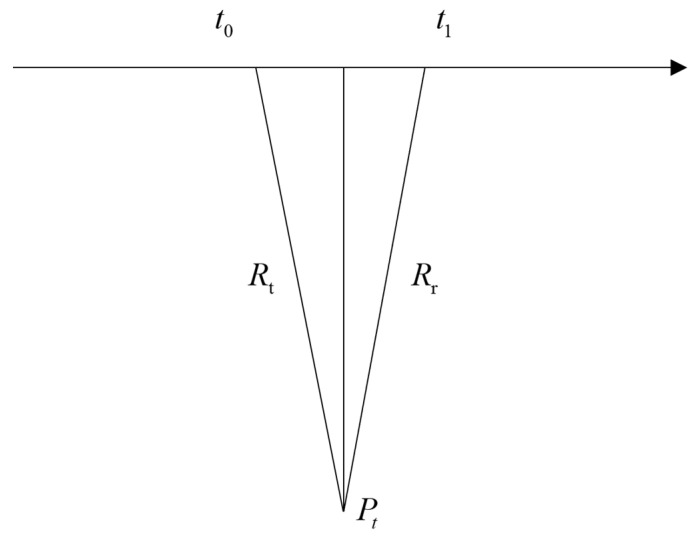
Spaceborne SAR positioning geometric model.

Figure 3 illustrates the positioning geometry of a spaceborne SAR. The satellite transmits a radar pulse at time $t_{0}$ and receives the corresponding echo at time $t_{1}$; the slant range of the transmit and receive propagation paths are denoted as $R_{t}$ and $R_{r}$, respectively. Owing to the separation between the transmit and receive phase centers, the signal propagation follows a bistatic geometry. According to the SAR imaging model, the range- Doppler equations are given by:

(25) $$\left\{\begin{array}{c} \left|P-P_{t}\left(t_{0}\right)\right|+\left|P-P_{r}\left(t_{1}\right)\right|=\left(R_{t}+R_{r}\right)\\ \frac{V_{t}\left(t_{0}\right)\cdot \left(P-P_{t}\left(t_{0}\right)\right)}{R_{t}}+\frac{V_{r}\left(t_{1}\right)\cdot \left(P-P_{r}\left(t_{1}\right)\right)}{R_{r}}=\lambda \cdot f_{d} \end{array}\right.$$

where the first equation represents the bistatic range constraint and the second equation corresponds to the Doppler constraint. Here, P denotes the position vector of the ground target, and $P_{t}\left(t_{0}\right)$ and $P_{r}\left(t_{1}\right)$ are the satellite position vectors at the transmission and reception time, respectively. $V_{t}\left(t_{0}\right)$ and $V_{r}\left(t_{1}\right)$ are the corresponding satellite velocity vectors. $f_{d}$ denotes the satellite Doppler centroid frequency, and λ is the radar wavelength.

Given the longitude and latitude coordinates of an isolated strong scattering point on the ground, its zero Doppler echo position in the azimuth-range domain is determined through an iterative solution. The procedure is described below:

First, the longitude and latitude coordinates of the ground scatterer are transformed into Earth-centered Earth-fixed (ECEF) coordinates.

The azimuth time is then initialized using the center of the current SAR image, which is assumed to correspond to the signal reception time $t_{1}$. Based on this initialization, the corresponding receive phase center position $P_{r}\left(t_{1}\right)$, velocity $V_{r}\left(t_{1}\right)$, and acceleration $A_{r}\left(t_{1}\right)$ are evaluated.

Using the ground point position and the receive phase center state at time $t_{1}$, the slant range R is calculated. The signal propagation delay between transmission and reception is then estimated as $∆t=\frac{2R}{c}$, where c is the speed of light.

The transmission time is subsequently determined as $t_{0}=t_{1}-∆t$. The corresponding transmit phase center position $P_{t}\left(t_{0}\right)$, velocity $V_{t}\left(t_{0}\right)$, and acceleration $A_{t}\left(t_{0}\right)$ are then obtained.

Based on the transmit and receive states, the azimuth time difference between the current row and the imaging time is computed:

(26) $$∆t=\frac{\frac{V_{t}\left(t_{0}\right)\cdot \left(P-P_{t}\left(t_{0}\right)\right)}{R_{t}}+\frac{V_{r}\left(t_{1}\right)\cdot \left(P-P_{r}\left(t_{1}\right)\right)}{R_{r}}-\lambda \cdot f_{d}}{\frac{\left(P-P_{t}\left(t_{0}\right)\right)\cdot A_{t}\left(t_{0}\right)-V_{t}\left(t_{0}\right)V_{t}\left(t_{0}\right)}{R_{t}}+\frac{\left(P-P_{r}\left(t_{1}\right)\right)\cdot A_{r}\left(t_{1}\right)-V_{r}\left(t_{1}\right)V_{r}\left(t_{1}\right)}{R_{r}}}$$

Using the updated value of ∆t, the transmission and reception times are refined iteratively. The iterative process continues until convergence is achieved, i.e., when the absolute variation $\left|∆t\right|$ becomes smaller than a predefined threshold. If the number of iterations exceeds a preset limit, the solution is considered invalid.

The predefined threshold represents the allowable azimuth time deviation between the geometrically predicted echo position and the observed signal peak. From a localization accuracy perspective, the threshold should be designed on the order of azimuth sample interval $T_{a}$, which corresponds to one azimuth pixel. This ensures that the activated control points maintain pixel-level positioning accuracy. During the calculating process, the search interval of ∆t must remain bounded to avoid ambiguity. Specifically, it should satisfy $T_{a}\leq ∆t\leq \frac{T_{scene}}{2}$, where $T_{scene}$ denotes the total azimuth time span of the imaged scene.

Once convergence is achieved, the azimuth position of the strong scatter is determined from the estimated azimuth time, and its range position is obtained from the corresponding slant range.

### 3.3. Inter-Channel Phase Error Estimation via Frequency Correlation Correlation

Taking into account the phase error between channels, the range–Doppler domain echo of the m-th receive channel can be expressed as

(27) $$S_{m}\left(\tau ,f_{a}\right)=\exp\left(j\phi_{m}\right)\cdot \exp\left(j2\pi f_{a}∆\eta_{m}\right)\cdot S_{0}\left(\tau ,f_{a}\right)$$

where $S_{0}\left(\tau ,f_{a}\right)$ denotes the reference channel echo, $\phi_{m}$ is the constant inter-channel phase error of the m-th channel relative to the reference channel, and $∆\eta_{m}$ represents the equivalent azimuth time delay caused by the displaced phase centers.

After activating an isolated strong scatterer using the geometric prediction described in Section 3.2, the echo signals of all channels are extracted in the vicinity of the zero-Doppler region. Each extracted signal is range-compressed and transformed into the Doppler domain. The frequency domain correlation between the m-th channel and the reference channel is then computed as

(28) $$\begin{array}{l} IN_{m}\left(f_{a}\right)=S_{m}\left(\tau ,f_{a}\right)\cdot S_{0}^{*}\left(\tau ,f_{a}\right)\\ =\exp\left(j\phi_{m}\right)\cdot \exp\left(j2\pi f_{a}∆\eta_{m}\right)\cdot {\left|S_{0}\left(\tau ,f_{a}\right)\right|}^{2} \end{array}$$

To suppress noise and residual clutter interference, an averaging operation is performed over a selected Doppler frequency interval δ around zero Doppler, yielding

(29) $$E_{f_{a}\in \delta }\left\{IN_{m}\left(f_{a}\right)\right\}=\exp\left(j\phi_{m}\right)\cdot \exp\left(-j2\pi f_{a}∆\eta_{m}\right)\cdot E\left\{{\left|S_{0}\left(\tau ,f_{a}\right)\right|}^{2}\right\}$$

The averaged correlation phase can be expressed as

(30) $$∠E_{f_{a}\in \delta }\left\{IN_{m}\left(f_{a}\right)\right\}=\phi_{m}+2\pi f_{a}∆\eta_{m}$$

Since the selected Doppler interval δ is centered at zero Doppler, ${\bar{f}}_{a}\approx 0$ and the second term can be effectively neglected. Consequently, the inter-channel phase error of the m-th channel can be estimated as:

(31) $$\phi_{m}=∠E_{f_{a}\in \delta }\left\{IN_{m}\left(f_{a}\right)\right\}$$

## 4. Spaceborne SAR Data Verification

To evaluate the practical effectiveness and robustness of the proposed phase error estimation method, a series of experiments were conducted using dual-channel spaceborne SAR datasets acquired by the LT1A, LT1B, and GF-3 satellites. These real-world datasets represent typical high-resolution wide-swath SAR acquisition scenarios and provide a valuable benchmark for assessing phase consistency across azimuth channels.

The key system parameters for the datasets including a radar operating wavelength of 0.23 m, antenna length of 9.8 m, satellite platform velocity of 7847.43 m/s, and Doppler bandwidth of 2400 Hz. The system operates with a pulse repetition frequency (PRF) of 1547.58 Hz, resulting in under-sampling in the azimuth direction for each individual channel, thereby requiring effective inter-channel phase calibration to enable correct signal reconstruction.

**Figure 4:**
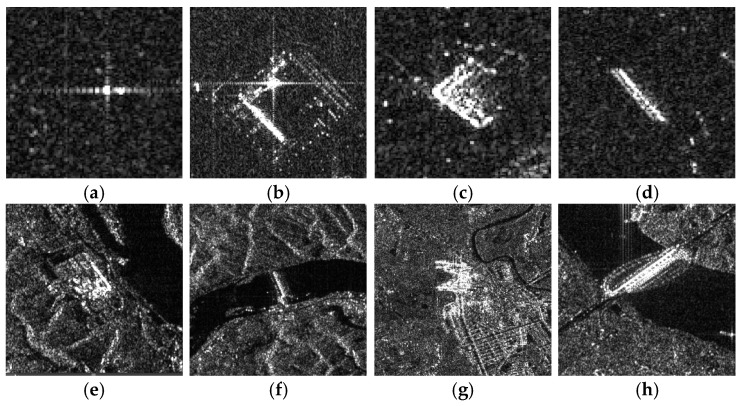
SAR image slices of isolated strong point targets containing strong backscattering targets, such as man-made metallic structures and calibration reflectors. (**a**) Corner reflector. (**b**) Trihedral corner reflector in buildings. (**c**) Building edges. (**d**) Dihedral corner reflector in buildings. (**e**) Isolated building in mountainous areas. (**f**) Simple bridge. (**g**) Transmission tower. (**h**) Cable-stayed bridge.

Based on the method described in Section 3, a set of isolated strong point targets were first extracted from the image domain using intensity-based filtering and spatial isolation constraints. Figure 4 presents representative SAR image slices that contain strong backscattering targets, such as man-made metallic structures and calibration reflectors, which are ideal for inter-channel calibration due to their high signal-to-clutter ratio.

**Figure 5:**
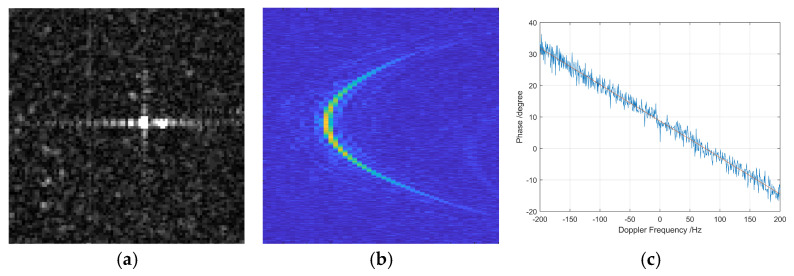
Multi-channel echo range migration curve of isolated strong point target. (**a**) Image slice amplitude. (**b**) Range migration curve after range compression in time-domain. (**c**) Channel phase error varying with Doppler frequency.

Figure 5 illustrates the multi-channel echo behavior and phase characteristics of a representative isolated strong point target, which is used to evaluate the proposed phase error estimation method.

In Figure 5a, a SAR image slice is presented, clearly showing the amplitude distribution of the scene. The isolated strong scatterer appears as a prominent, spatially distinct high-intensity point target, standing out from the surrounding background. This confirms its suitability as a calibration reference, given its high signal-to-clutter ratio and limited interference from neighboring scatterers. Such targets are critical to ensure the reliability of frequency domain correlation-based estimation, as they provide well-defined and consistent echo responses across channels.

Figure 5b depicts the range migration curves of the same target after time-domain range compression. The aligned trajectories of the echoes from multiple azimuth channels indicate that the proposed range–Doppler positioning model successfully located the echo centers in both range and azimuth dimensions. The high degree of overlap among the range profiles demonstrates consistent signal propagation and reception across the channels, laying the foundation for meaningful phase comparison in the frequency domain. This step also verifies the effectiveness of the isolated point echo extraction process, which is essential for isolating the low-Doppler components required by the algorithm.

Figure 5c presents the estimated channel phase error as a function of Doppler frequency, computed via conjugate multiplication of the Doppler-transformed signals. It is observed that the phase error exhibits relatively low fluctuation within the low-Doppler region (centered near zero), where signal coherence is strongest and ambiguity is minimal. This observation is consistent with theoretical expectations, as the low-Doppler components correspond to backscatter near the nadir direction with minimal geometric distortion. The curve also helps identify the valid frequency band that can be used for robust estimation, while avoiding regions with aliasing or phase instability.

These subfigures show the performance of the core components of the proposed method, including strong target extraction, accurate echo localization, and Doppler-domain phase calibration. The results indicate that the algorithm is effective for real SAR data and show that reliable channel phase error estimation can be achieved in an automated and data-driven manner.

**Figure 6:**
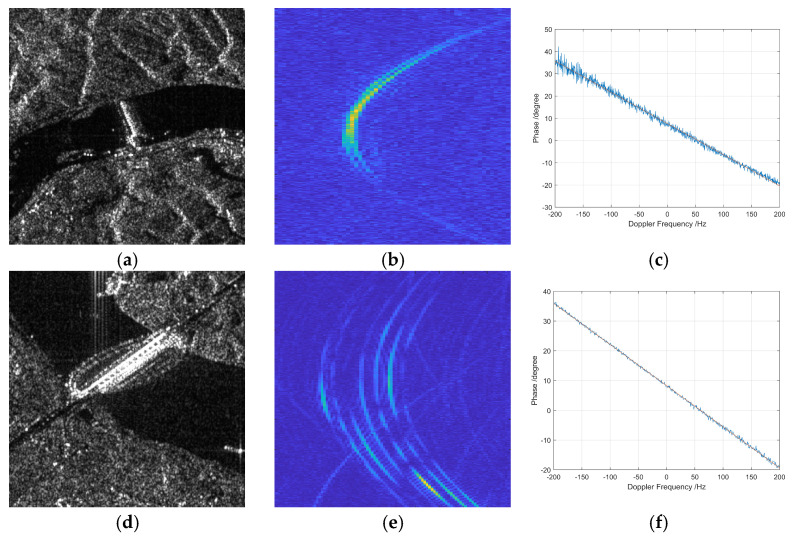
Multi-channel echo range migration curves of a man-made bridge target. (**a**,**d**) Image slice amplitudes; (**b**,**e**) Range migration curves after time-domain range compression; (**c**,**f**) Channel phase errors as functions of Doppler frequency.

Figure 6 presents the multi-channel echo characteristics of a man-made bridge target, which serves as a typical extended strong scatterer to further evaluate the performance of the proposed phase error estimation method under complex spatial structures.

In Figure 6a,d, SAR image slices centered on two different bridge targets are shown. The bridges appear as elongated high-intensity structures with clear backscattering contrast against the surrounding terrain, suggesting the presence of dominant scatterers such as metallic beams and support structures. Although less spatially isolated than ideal point targets, such man-made objects provide valuable calibration references due to their high radar reflectivity and structural regularity.

Figure 6b,e display the range migration curves for multi-channel echoes of the two bridge targets after time-domain range compression. The curves from different channels remain closely aligned along the azimuth dimension, indicating consistent echo localization and range delay modeling across the channels. This further validates the accuracy of the range–Doppler-based echo positioning process, even in cases where the target is extended rather than point-like.

In Figure 6c,f, the estimated inter-channel phase errors are plotted as a function of Doppler frequency. The curves show that the phase difference between channels remains relatively stable in the low-Doppler region, which corresponds to the center of the beam footprint. The results confirm that even in the presence of extended structural scattering, the proposed frequency domain correlation method can yield reliable and consistent phase error estimates, as long as the target contains concentrated scattering centers with sufficient coherence.

Overall, this figure demonstrates that the proposed method is applicable not only to ideal point targets but also to larger, complex man-made structures. The ability to maintain phase estimation accuracy in such scenarios highlights the robustness and generalizability of the approach, particularly for real-world calibration tasks in heterogeneous urban or infrastructural environments.

**Figure 7:**
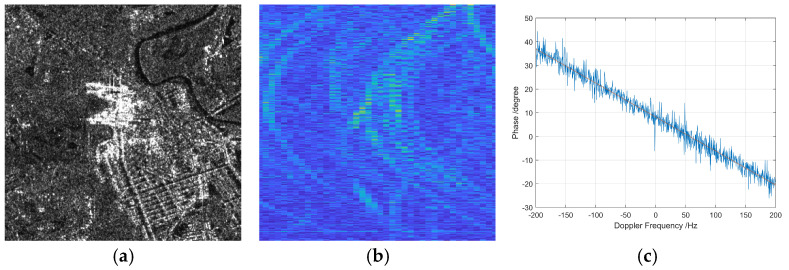
Multi-channel echo range migration curve of power transmission tower. (**a**) Image slice amplitude. (**b**) Range migration curve after range compression in time-domain. (**c**) Channel phase error varying with Doppler frequency.

Figure 7 demonstrates the multi-channel echo range migration behavior observed from a power transmission tower target in a synthetic aperture radar (SAR) imaging system. In Figure 7a, the image slice amplitude is displayed, representing the spatial distribution of reflected signal strength from the tower across multiple radar channels. This amplitude map provides a visual insight into the scattering characteristics and geometric structure of the target, with stronger reflections corresponding to key structural components such as insulators and tower arms.

Figure 7b shows the range migration curve after applying range compression in the time domain. Range compression serves to improve range resolution by correlating the received echo with the transmitted pulse, effectively compressing the echo in time and concentrating the target energy into a narrow peak. The resulting curve describes the echo’s range trajectory as a function of Doppler frequency, revealing the range cell migration driven by both the radar platform’s motion relative to the target and the spatial diversity across multiple channels.

Figure 7c depicts the variation in channel phase errors with Doppler frequency. These phase errors arise from system imperfections, platform motion errors, and channel mismatches, and they vary nonlinearly with Doppler frequency. Accurate estimation and correction of these phase errors are essential because uncorrected phase distortions lead to image defocusing and degradation of target feature extraction.

Together, these three subfigures provide a comprehensive view of the multi-channel echo characteristics for the power transmission tower, highlighting the interplay between amplitude distribution, range migration behavior, and phase error dynamics. This analysis is crucial for refining multi-channel SAR signal processing algorithms, ensuring accurate target detection, imaging, and classification in complex scenes involving man-made structures.

**Figure 8:**
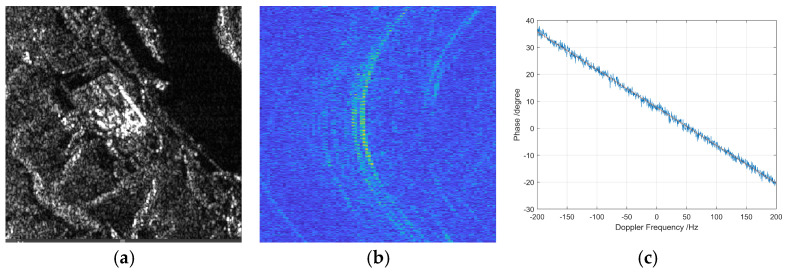
Multi-channel echo range migration curve of single house in the mountains. (**a**) Image slice amplitude. (**b**) Range migration curve after range compression in time-domain. (**c**) Channel phase error varying with Doppler frequency.

Figure 8 presents the multi-channel echo range migration characteristics of a single house located in a mountainous area. Figure 8a depicts the image slice amplitude, illustrating the spatial distribution of reflected radar energy from the house and its surrounding terrain. The amplitude highlights key scattering features such as the roof, walls, and nearby topographical variations, which contribute to the unique backscatter signature of the house in a complex mountainous environment.

Figure 8b shows the range migration curve obtained after range compression in the time domain. The range compression process sharpens the echo response, enabling finer range resolution and clearer delineation of the house structure despite the uneven terrain and possible multipath effects.

Figure 8c illustrates the channel phase error variation with Doppler frequency across the multi-channel system. Phase errors arise from sensor platform instabilities, channel mismatches, and complex scattering mechanisms influenced by the mountainous environment. The phase error curve indicates the degree and pattern of phase distortion that must be estimated and compensated to achieve precise image focusing.

Overall, this figure comprehensively captures the challenges and characteristics of multi-channel SAR echoes from a single house in mountainous terrain, emphasizing the importance of accurate range migration compensation and phase error correction in high-resolution SAR imaging of complex natural and man-made features.

Table 1 summarizes the channel phase error estimation results for each point across different satellite scenes. The data is organized by satellite identifier, scene ID, and point ID, presenting the channel phase error in degrees for each measurement. For each group of points within a scene, the mean and standard deviation of the phase errors are calculated to reflect the central tendency and variability of the channel phase error.

For satellite LT1A in Scene 1, five points are listed with phase errors ranging approximately from 16.5° to 17.6°, resulting in a mean phase error of 16.89° and a standard deviation of 1.31°, indicating a relatively consistent but slightly variable phase distortion across the measured points. Satellite LT1B shows two scenes: Scene 2 with five points exhibits phase errors centered around 8.83° (standard deviation 1.18°), while Scene 3 with ten points has a slightly lower mean phase error of 7.94° and a tighter variability (standard deviation 0.81°), suggesting more stable phase conditions in this scene.

This tabulated data is critical for understanding and quantifying phase errors that arise in multi-channel SAR systems. Accurate phase error estimation and characterization enable effective compensation during signal processing, which directly influences the focusing quality and image clarity. The relatively low standard deviations across points within each scene imply a manageable level of phase error consistency, facilitating targeted calibration strategies tailored to each satellite and scene environment.

**Figure 9:**
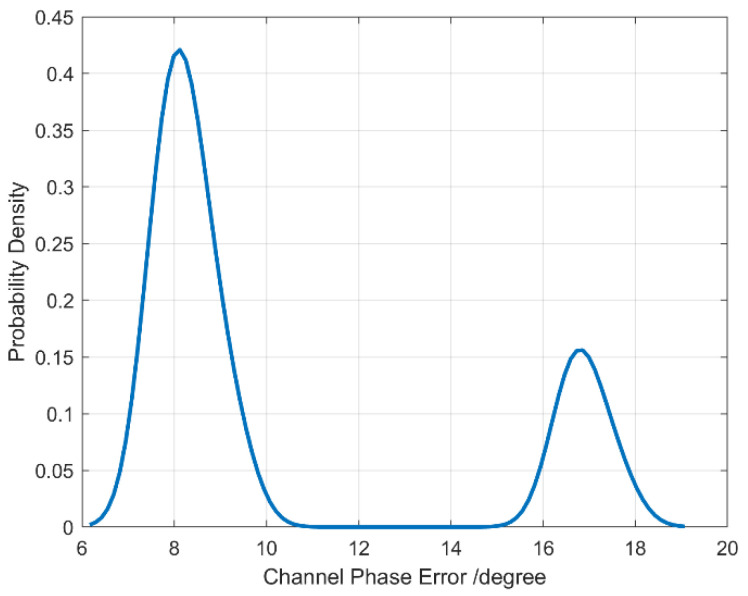
Kernel density function of phase error estimation results.

Figure 9 displays the kernel density estimation (KDE) of the phase error values obtained from multi-channel SAR data processing. The KDE curve provides a smooth, continuous approximation of the underlying probability distribution of the phase errors across all measured points and scenes. By visualizing the phase error distribution in this way, the figure reveals the central tendency, spread, and modality of the phase errors without being confined to discrete histogram bins.

By visualizing the distribution of phase errors, the density plot highlights their most probable values during calibration: peaks in the KDE represent frequently occurring magnitudes, while the tails capture rarer, significant deviations. This analysis is thus essential for assessing phase stability and developing robust correction algorithms.

This visualization helps identify whether the phase errors follow a unimodal, symmetric distribution centered around a specific value or exhibit multiple modes, skewness, or outliers, which would suggest more complex phase behavior. Overall, the kernel density function provides a comprehensive statistical insight into the phase error characteristics, supporting improved signal processing and imaging accuracy in multi-channel SAR systems.

**Figure 10:**
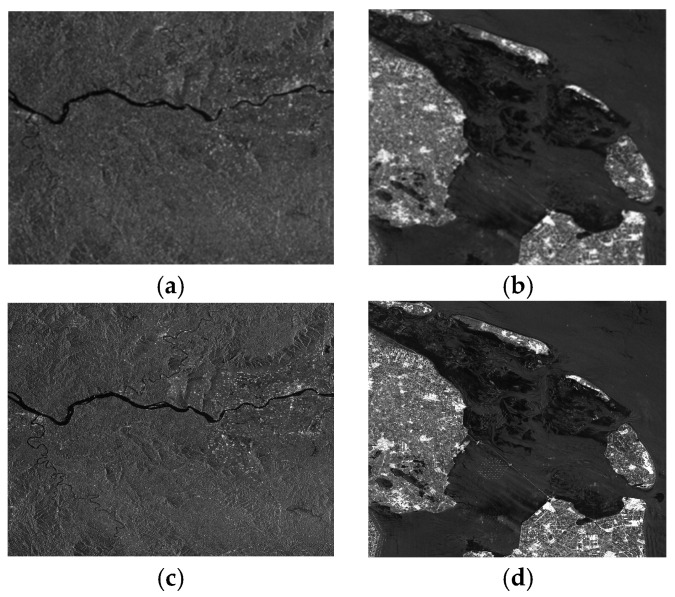
Comparison of SAR imaging results before and after inter-channel phase error correction. (**a**) Uncalibrated image of a mountainous area; (**b**) Uncalibrated image of a sea–land border area; (**c**) Corrected image of the mountainous area; (**d**) Corrected image of the sea–land border area.

To evaluate the performance of the proposed framework, Figure 10 provides a side-by-side comparison between the uncalibrated and corrected SAR images across two representative scenes. Figure 10a,c display a mountainous region, where the uncalibrated image is prone to severe defocusing and azimuth ambiguities caused by inter-channel phase inconsistencies. After applying the proposed correction, as shown in Figure 10c, the image quality is significantly improved. The ridge lines, slopes, and structural targets (such as isolated buildings) are more distinctly outlined, demonstrating the suppression of inter-channel phase errors and the restoration of fine geometric details.

Furthermore, Figure 10b,d illustrate a sea–land border area, which is typically characterized by high contrast and complex scattering. In Figure 10b, the coastline and land-based structures are blurred by striping artifacts and ghosting effects resulting from phase mismatches. Following the calibration, Figure 10d exhibits sharper coastline boundaries and better-defined roads and infrastructure. Notably, the sea surface becomes smoother and more uniform as the noise and ambiguities are effectively reduced. These improvements are crucial for maritime surveillance and coastal monitoring, where high-fidelity imaging is essential for accurate target detection and feature extraction.

**Figure 11:**
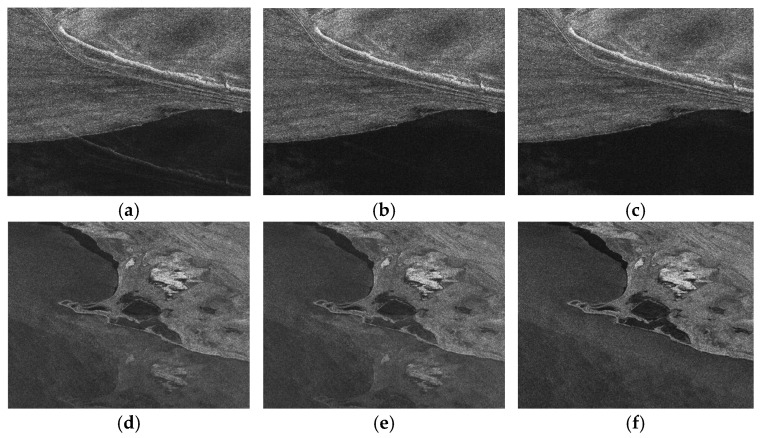
GF-3 spaceborne SAR images before and after inter-channel phase error correction in two different scenes. (**a**,**d**) Without channel phase correction; (**b**,**e**) With channel phase correction using the orthogonal subspace method; and (**c**,**f**) With channel phase correction using the proposed frequency-domain correlation method.

Figure 11 provides a direct visual comparison of GF-3 SAR imagery before and after inter-channel phase error correction. As illustrated in Figure 11a,d, uncorrected images suffer from distinct azimuth blurring and defocusing artifacts, which are directly attributable to phase inconsistencies across channels. Figure 11b,e show that the orthogonal subspace method restores image sharpness to a certain extent; yet, residual phase errors induce subtle geometric distortions and the loss of fine structural details, a phenomenon particularly pronounced in scenes with sparse land coverage. In contrast, Figure 11c,f clearly demonstrate that the proposed frequency-domain correlation method achieves robust correction of inter-channel phase errors. This results in well-defined target outlines, superior image focusing, and markedly enhanced radiometric consistency across the entire scene.

Table 2 presents a comparison of inter-channel phase errors estimated from GF-3 SAR data by the subspace-based method and the proposed frequency-domain correlation method. Results are summarized for three representative scene types: urban and agricultural, mountainous, and coastal areas.

As shown in Table 2, both the subspace-based method and the proposed method produce comparable estimates in urban/agricultural and mountainous scenes. However, in the coastal region, where scattering conditions are more complex, the subspace-based method exhibits significant deviations, whereas the proposed method maintains stable and physically reasonable estimates. These results indicate that the proposed method provides improved robustness under challenging scattering environments, rather than uniformly enhanced consistency across all scatterer types.

In the mountainous area, where the dominant scattering mechanisms are primarily volume scattering combined with terrain-induced geometric effects, the estimated parameters remain stable and consistent across different methods.

**Figure 12:**
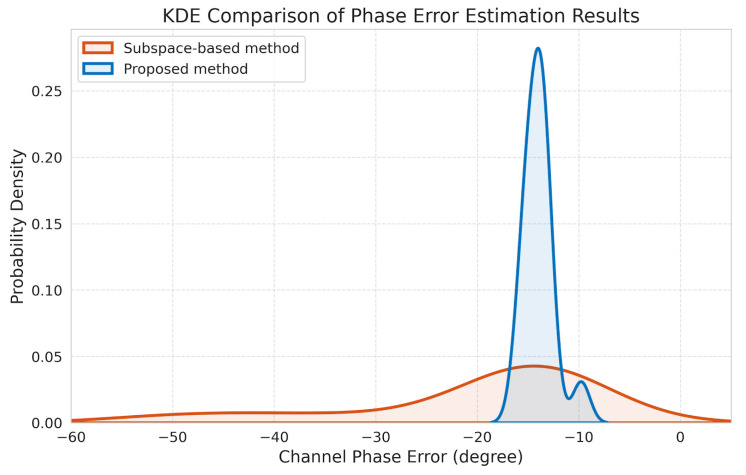
Kernel density estimation (KDE) comparison of inter-channel phase error estimation results between the subspace-based method and the proposed method using GF-3 datasets.

To provide a clearer quantification of the proposed improvement, Figure 12 illustrates the probability density distribution of the estimation results. As observed in the figure, the proposed geometry-driven frequency-domain correlation method (blue solid line) exhibits exceptional estimation consistency. Its probability density is highly concentrated around −14°, characterized by a sharp and narrow-bandwidth unimodal peak. In contrast, the distribution of the state-of-the-art orthogonal subspace-based method (red dashed line) is significantly more scattered, featuring prominent tails and discrete peaks between −30° and −60°. This divergence is primarily due to the subspace method’s difficulty in obtaining an accurate covariance matrix estimate in complex scattering environments, such as coastal regions, resulting in severe estimation biases. These comparative results strongly demonstrate that the proposed framework possesses superior stability, robustness, and high precision across diverse imaging scenarios. It performs particularly well in heterogeneous environments, such as sea–land border areas, where conventional methods frequently fail to maintain reliable performance.

In the coastal scene, the subspace-based method shows large fluctuations and outliers (e.g., −33°, −50°, −43°) due to strong mixed land–sea scattering, which the covariance matrix cannot be accurately estimated. In contrast, the proposed method effectively suppresses these deviations, maintaining phase errors within a stable range around −14°, which demonstrates its robustness and adaptability in complex environments.

Together, these corrected images validate the effectiveness of phase error calibration in diverse environments. They highlight how improved coherence across channels enhances target definition and structural clarity, enabling more reliable scene interpretation and follow-on applications such as classification and change detection.

The removal of phase errors reduces blurring and ghosting artifacts that typically degrade image resolution and hinder target discrimination. The resulting improvement in imaging fidelity renders structural details—both man-made (e.g., buildings, power towers) and natural (e.g., mountainous terrain)—more distinct and well-defined. This directly supports enhanced performance in target detection, classification, and subsequent interpretation tasks.

Overall, the experimental results on spaceborne SAR data validate the automation, accuracy, and generalization capability of the proposed method. In contrast to conventional methods, the proposed framework relies on a globally built isolated strong scatterer library and geometric modeling rather than manual point selection or scene-specific tuning, thereby ensuring scalable deployment across diverse imaging conditions and platform configurations. The proposed method is well suited for practical onboard calibration systems and large-scale post-processing pipelines in next-generation HRWS SAR missions.

## 5. Discussion

The experimental results demonstrate that the proposed geometry-driven phase error estimation method is effective and robust when applied to real spaceborne azimuth multi-channel SAR datasets. This section discusses the key strengths of the proposed approach in comparison with traditional methods, analyzes its limitations and scenario adaptability, and examines practical challenges associated with deployment in operational SAR systems.

### 5.1. Key Strengths and Practical Impact Relative to Conventional Methods

Compared with conventional inter-channel phase error estimation techniques, the proposed method exhibits several distinctive advantages that significantly enhance its practical applicability in operational SAR systems.

Firstly, the method achieves a high degree of automation, addressing a critical limitation of traditional approaches. Existing phase error estimation techniques typically rely on manual selection of strong scatterers or scene-dependent parameter tuning. Such procedures are labor-intensive, subjective, and difficult to scale for large-volume SAR data processing. By constructing a global radar landmark control point library and leveraging a geometric echo prediction model, the proposed method enables automatic activation of suitable control points without manual intervention, thereby improving efficiency and reducing human-induced errors.

Secondly, the proposed framework introduces a geometry-driven estimation paradigm. Instead of extracting reference targets directly from SAR images, the method predicts echo positions using prior geometric information and platform parameters. This decouples phase error estimation from scene-specific image characteristics and enhances robustness in heterogeneous environments.

Thirdly, the frequency-domain correlation strategy employed in the proposed method focuses on low-Doppler components with high coherence, resulting in stable and statistically consistent phase estimates across different sensors and acquisition conditions. This property is particularly advantageous for azimuth multi-channel SAR systems operating under under-sampled conditions, where phase consistency is critical for high-quality signal reconstruction.

### 5.2. Limitations and Scenario Adaptability

Despite these strengths, the proposed method has inherent limitations that restrict its applicability in certain scenarios. The primary constraint lies in its dependence on the availability of isolated strong scatterers within the imaged scene. In clutter-dominated environments, such as dense vegetation, rough terrain, or homogeneous distributed targets, the number of usable control points may be significantly reduced.

In such cases, the estimation accuracy and robustness may degrade due to insufficient high-coherence reference signals. To address this limitation, future research should explore hybrid strategies that integrate the proposed geometry-driven framework with subspace-based or statistical estimation techniques, enabling complementary utilization of distributed scatterers when isolated point targets are sparse.

In addition, it should be emphasized that the proposed phase-error estimation framework assumes stationary or quasi-stationary strong scatterers with stable scattering characteristics. Moving or rotating targets generally do not satisfy this condition due to time-varying scattering mechanisms and Doppler frequency shifts. Therefore, the method is not intended to directly estimate inter-channel phase errors caused by target motivation.

However, since inter-channel phase errors are system-induced, they can be reasonably assumed to be scene-invariant within a single acquisition. As a result, phase calibration derived from stationary scatterers remains valid for moving-target signals. In this context, the proposed framework can be applicable in dynamic scenes, provided that reliable stationary references are available for calibration.

### 5.3. Practical Implementation Considerations in Operational SAR Systems

Although the proposed method has been validated using real spaceborne SAR data, its deployment in operational systems introduces additional challenges that must be carefully addressed.

A critical prerequisite for reliable phase error estimation is the validation of activated radar landmarks. Each isolated strong scatterer must be rigorously verified before being used as a control point. In operational environments, scatterer identification errors primarily manifest as false alarms and missed detections. False identification of non-isolated targets may introduce distorted echo boundaries due to interference from adjacent clutter or nearby scatterers, leading to biased phase estimates.

Moreover, residual geometric modeling errors, orbit inaccuracies, and platform attitude uncertainties may affect echo position prediction accuracy. These factors impose practical constraints on control point activation reliability and highlight the importance of incorporating robust validation mechanisms and error tolerance strategies in system implementation.

Addressing these challenges is essential for transitioning the proposed method from experimental validation to fully operational SAR processing pipelines.

## 6. Conclusions

In this paper, an automated frequency-domain correlation method for inter-channel phase error estimation in spaceborne azimuth multi-channel SAR systems has been proposed. By introducing a global radar landmark prior, the proposed method establishes a geometry-driven and scene-independent framework for phase error estimation. This approach shifts the paradigm from image-dependent target selection to geometric-prior-based control point activation.

Extensive experiments conducted on real spaceborne SAR datasets demonstrate that the proposed method achieves high estimation accuracy, strong stability, and low variability across different sensors and imaging scenarios. Accurate compensation of the estimated phase errors substantially enhances image focusing and mitigates azimuth ambiguities.

The proposed approach is particularly suitable for operational SAR systems, as it eliminates manual intervention and exhibits strong adaptability to diverse imaging environments. Future work will focus on incorporating echo modeling techniques and machine learning-based scatterer recognition methods, to enhance robustness in clutter-rich and structurally complex scenes.

## Figures and Tables

**Table 1:** Channel phase error estimation result of each point.

Satellite	Scene ID	Point ID	Channel Phase Error (Degree)	Mean	Standard Deviation (3σ)
LT1A	1	1	16.9136	16.8945	1.3053
		2	16.5007		
		3	16.5282		
		4	16.9543		
		5	17.5757		
LT1B	2	1	8.7094	8.8317	1.1816
		2	9.0798		
		3	9.3709		
		4	8.3724		
		5	8.6259		
	3	1	8.0672	7.9433	0.8096
		2	7.6637		
		3	8.2901		
		4	7.6651		
		5	7.7850		
		6	7.6910		
		7	7.6766		
		8	8.1872		
		9	8.2725		
		10	8.1344		

**Table 2:** GF-3 SAR inter-channel phase error estimation results for different scene types, and comparison between the subspace-based method and the proposed method.

Scene Type	Point ID	Channel Phase Error Using Subspace-Based Method (Degree)	Channel Phase Error Using Proposed Method (Degree)
Urban and agricultural area	1	−13.7988	−13.6394
	2	−13.9497	−15.3965
	3	−13.8652	−14.3911
	4	−13.6569	−13.1286
	5	−15.2777	−16.0555
Mountainous area	1	−11.1715	−12.7589
	2	−13.6999	−13.6160
	3	−14.5593	−13.3329
	4	−14.8844	−13.8393
	5	−15.3969	−14.8127
Coastal area	1	−33.6073	−14.3035
	2	−13.3435	−13.8055
	3	−50.6111	−9.77991
	4	−42.7976	−14.9864
	5	−23.0978	−15.2865

## Data Availability

Data are contained within the article.

## References

[1] Huber S., de Almeida F.Q., Villano M., Younis M., Krieger G., Moreira A. (2018). Tandem-L: A technical perspective on future spaceborne SAR sensors for Earth observation. IEEE Trans. Geosci. Remote Sens..

[2] Xu G., Zhang B., Yu H., Chen J., Xing M., Hong W. (2022). Sparse synthetic aperture radar imaging from compressed sensing and machine learning: Theories, applications and trends. IEEE Geosci. Remote Sens. Mag..

[3] Kim J.H., Younis M., Prats-Iraola P., Krieger G., Moreira A. (2013). First spaceborne demonstration of digital beamforming for azimuth ambiguity suppression. IEEE Trans. Geosci. Remote Sens..

[4] Ni P., Xu G., Pei H., Qiao Y., Yu H., Hong W. (2025). Dual-stream manifold multiscale network for target recognition in complex-valued SAR images with electromagnetic feature fusion. IEEE Trans. Geosci. Remote Sens..

[5] Zhou H., Xu G., Xia X., Li T., Yu H., Liu Y. (2025). Enhanced matrix completion method for superresolution tomography SAR imaging: First large-scale urban 3-D high-resolution results of LT-1 satellites using monostatic data. IEEE J. Sel. Top. Appl. Earth Obs. Remote Sens..

[6] Li S.Q., Yang R.L. (2004). Error analysis of displaced phase centers multiple azimuth beam synthetic aperture radar. Acta Electron. Sin..

[7] Zhang L., Xing M.D., Qiu C.W., Bao Z. (2010). Adaptive two-step calibration for high-resolution and wide-swath SAR imaging. IET Radar Sonar Navig..

[8] Gebert N., Krieger G., Moreira A. (2005). SAR signal reconstruction from non-uniform displaced phase centre sampling in the presence of perturbations. IGARSS 2004. 2004 IEEE International Geoscience and Remote Sensing Symposium.

[9] Zhang L., Zhang N., Gao Y., Wang K., Liu X. (2016). Reconstruction of azimuth signal for multichannel HRWS SAR imaging based on periodic extension. 2016 IEEE International Geoscience and Remote Sensing Symposium (IGARSS), Beijing, China, 10–15 July 2016.

[10] Zhang L., Gao Y., Wang K., Liu X. (2017). Azimuth signal reconstruction for HRWS SAR from recurrent nonuniform samples. 2016 CIE International Conference on Radar (RADAR), Nanjing, China, 10–13 October 2016.

[11] Cheng P., Wan J., Xin Q., Wang Z. (2016). Multichannel azimuth reconstruction of high-resolution wide-swath SAR via Vandermonde matrix. 2016 IEEE International Geoscience and Remote Sensing Symposium (IGARSS), Beijing, China, 10–15 July 2016.

[12] Cheng P., Wan J., Xin Q., Wang Z., He M., Nian Y. (2017). An improved azimuth reconstruction method for multichannel SAR using Vandermonde matrix. IEEE Geosci. Remote Sens. Lett..

[13] Liu Y.Y., Li Z.F., Suo Z.Y., Bao Z. (2012). A novel channel phase bias estimation method for spaceborne along-track multi-channel HRWS SAR in time-domain. IET International Radar Conference 2013.

[14] Gebert N., Krieger G. (2009). Azimuth phase center adaptation on transmit for high-resolution wide-swath SAR imaging. IEEE Geosci. Remote Sens. Lett..

[15] Zhang S.X., Xing M.D., Xia X.G., Liu Y.Y., Guo R., Bao Z. (2013). A robust channel-calibration algorithm for multichannel azimuth HRWS SAR imaging based on local maximum-likelihood weighted minimum entropy. IEEE Trans. Image Process..

[16] Li Z., Bao Z., Wang H., Liao G. (2006). Performance improvement for constellation SAR using signal processing techniques. IEEE Trans. Aerosp. Electron. Syst..

[17] Zhou L., Deng M., He J., Wang B., Zhang S., Liu X., Wei S. (2024). A HRWS SAR motion compensation method with multichannel phase correction. Remote Sens..

[18] Weiss A.J., Friedlander B. (1990). Eigenstructure methods for direction finding with sensor gain and phase uncertainties. Circuits Syst. Signal Process..

[19] Liu Q., Ye Z., Zhu C., Ouyang D., Gu D., Wang H. (2025). Intelligent target detection in synthetic aperture radar images based on multi-level fusion. Remote Sens..

[20] Bai L., Xu W., Huang P., Tan W., Qi Y., Chen Y., Gao Z. (2024). Phase noise compensation algorithm for space-borne azimuth multichannel SAR. Sensors.

[21] Xu Z., Lu P., Cai Y., Li J., Yang T., Wu Y., Wang R. (2023). An efficient channel imbalance estimation method based on subadditivity of linear normed space of sub-band spectrum for azimuth multichannel SAR. Remote Sens..

[22] Xu W., Bai L., Huang P., Tan W., Dong Y. (2024). A space-borne SAR azimuth multi-channel quantization method. Electronics.

[23] Jiang N., Du H., Ge S., Zhu J., Feng D., Wang J., Huang X. (2023). High-resolution azimuth missing data SAR imaging based on sparse representation autofocusing. Remote Sens..

[24] Xu Z., Lu P., Cai Y., Wu Y., Wang R. (2023). Performance analysis of channel imbalance control and azimuth ambiguity suppression in azimuth dual receiving antenna mode of LT-1 spaceborne SAR system. Remote Sens..

[25] Cai Y., Deng Y., Zhang H., Wang R., Wu Y., Cheng S. (2022). An image-domain least L1-norm method for channel error effect analysis and calibration of azimuth multi-channel SAR. IEEE Trans. Geosci. Remote Sens..

[26] Huang H., Huang P., Liu X., Xia X.G., Deng Y., Fan H., Liao G. (2022). A novel channel errors calibration algorithm for multichannel high-resolution and wide-swath SAR imaging. IEEE Trans. Geosci. Remote Sens..

[27] Liu Y., Li Z., Wang Z., Bao Z. (2014). On the baseband Doppler centroid estimation for multichannel HRWS SAR imaging. IEEE Geosci. Remote Sens. Lett..

[28] Xiao F., Ding Z., Li Z., Long T. (2020). Channel error effect analysis for reconstruction algorithm in dual-channel SAR imaging. IEEE Geosci. Remote Sens. Lett..

[29] Gao C.G., Deng Y.K., Feng J. (2011). Theoretical analysis on the mismatch influence of displaced phase center multiple-beam SAR systems. J. Electron. Inf. Technol..

[30] Sun G.C., Xiang J., Wang Y., Zhang Z., Yang J., Xing M., Bao M., Bao Z. (2022). A postmatched-filtering image-domain subspace method for channel mismatch estimation of multiple azimuth channels SAR. IEEE Trans. Geosci. Remote Sens..

[31] Guo X., Gao Y., Wang K., Liu X. (2016). Improved channel error calibration algorithm for azimuth multichannel SAR systems. IEEE Geosci. Remote Sens. Lett..

[32] Zhang L., Gao Y., Liu X. (2017). Robust channel phase error calibration algorithm for multichannel high-resolution and wide-swath SAR imaging. IEEE Geosci. Remote Sens. Lett..

[33] Liang D., Wang R., Deng Y., Fan H., Zhang H., Zhang L., Wang W., Zhou Y. (2019). A channel calibration method based on weighted backprojection algorithm for multichannel SAR imaging. IEEE Geosci. Remote Sens. Lett..

[34] Xu Y., Zhang F., Chen L., Wan Y., Jiang T. (2025). A Novel Error Correction Method for Airborne HRWS SAR Based on Azimuth-Variant Attitude and Range-Variant Doppler Domain Pattern. Remote Sens..

[35] Zhang M., Meng Z., Wang G., Xue Y. (2024). Range-Dependent Channel Calibration for High-Resolution Wide-Swath Synthetic Aperture Radar Imagery. Sensors.

[36] Qiu J., Zhang Z., Deng Y., Zhang H., Wang W., Chen Z., Hou S., Feng Y., Wang N. (2025). A Novel Real-Time Multi-Channel Error Calibration Architecture for DBF-SAR. Remote Sens..

[37] Kumar A., Giusti E., Martorella M. (2015). Inverse SAR Pulse-to-Pulse Phase Correction for Improving SAR Imaging of Non-Cooperative Targets. 2025 IEEE Radar Conference (RadarConf25), Krakow, Poland, 4–10 October 2025.

[38] Giusti E., Kumar A., Martorella M. (2015). A Novel Back-Projection Based ISAR Approach for Non-Cooperative Moving Targets in SAR Images. 2025 IEEE Radar Conference (RadarConf25), Krakow, Poland, 4–10 October 2025.

[39] Martorella M., Berizzi F., Haywood B. (2005). Contrast maximisation based technique for 2-D ISAR autofocusing. IEE Proc.-Radar Sonar Navig..

[40] Carrara W., Goodman R., Majewski R. (1995). Spotlight Synthetic Aperture Radar: Signal Processing Algorithms.

